# Are toe fringes important for lizard burying in highly mobile sand?

**DOI:** 10.1186/s12983-024-00546-y

**Published:** 2024-09-30

**Authors:** Peng Zheng, Tao Liang, Lei Shi

**Affiliations:** 1 Key Laboratory of Ecological Adaptation and Evolution of Extreme Environment Biology in Xinjiang, College of Life Sciences, Xinjiang Agricultural University, Ürümqi, 830052 Xinjiang China; 2 Tel Aviv University, 69978 Tel Aviv, Israel

**Keywords:** Functional traits, Reptiles, Sand burial performance, Substrate properties

## Abstract

Toe fringes are a key innovation for sand dwelling lizards, and the relationship between toe fringe function and substrate properties is helpful in understanding the adaptation of lizards to sand dune environments. We tested the sand burial performance of *Phrynocephalus mystaceus* on different sand substrates with toe fringe manipulation, with the aim of assessing whether the function of the toe fringes shifts under different substrate properties, especially in highly mobile substrates. The sand burial performance of *P. mystaceus* was influenced by substrate properties in relation to the toe fringe states of the lizard. After removal of the bilateral toe fringes, the sand burial ability score of *P. mystaceus* was significantly higher on sand substrates below 100 mesh than on native sand substrates. As the angle of stability of the substrate properties decreased, the sand burial performance of the lizard was even better after the bilateral toe fringes were removed. The results of the LASSO model and the path analysis model showed that the stability angle provided the opposite effect on sand burial performance in different toe fringe states. These results further suggest that the sand burial function of toe fringes may not be suitable for highly mobile sand substrates. It remains to be tested further whether the function of toe fringes is more important for running on sand.

**Supplementary Information:**

The online version contains supplementary material available at 10.1186/s12983-024-00546-y.

## Background

Body size and shape play fundamental roles in the functioning of an organism. Comparisons of locomotor and dietary groups highlight key differences in body proportions that may mechanistically underlie the occupation of major ecological niches [1, 2]. The relationship between morphology and function is frequently linked to ecological factors. Some anti-predatory behaviours using ecological substrates, such as burial in sand, provide an opportunity to explore the links between form, function, and ecology [3]. Burial in sand is a complex anti-predatory behaviour associated with several key factors, such as predation pressure [4], escape distance, and dune location [5]. The long bodies with short limbs are more conducive to "terrestrial swimming" in lizard (*Brachymeles* and *Lerista*) that live in the soil [6]. This may be related to the way lizards move within the substrate, such as the desert-dwelling sandfish (*Scincus scincus*) moves within dry sand [7]. The expression of sand-burying behaviour varies among different species of lizards, e.g. The agamids, *Phrynocephalus* and *Agama etoshae* employ lateral body oscillations and descend vertically, the scincids, gerrhosaurids and *Scincus*, *Angolosaurus*, both use high amplitude sinusoidal movements of the body and tail [8].

Two morphological adaptations were proposed for sand swimming, namely a cylindrical body with short limbs and a blunt snout [8]. A flattened body with well-developed limbs and toe fringes is more suitable for burial in sand [8, 9] (Table S1). Toe fringes were first thought to be an adaptation of lizards to the movement of drifting sand [10]. Toe fringes also promote the ability of lizards to burial in sand and conserve physical energy [3]. Variation in the morphology of the toe fringes, a typical morphological structural feature adapted to desert environments, shows a strong correlation with the substrate type [8]. There are four types of fringes, namely triangular, projectional, conical, and rectangular fringes [11]. The results of the functional analysis of toe fringes showed that the rectangular fringes provide more effective surface area for the same area when locomotion on water compared to the other three denticulate fringes, while the denticulate fringes (triangular, projectional, and conical fringes) provide a larger effective surface area on sand. Thus, triangular, projectional, and conical fringes are associated with sandy landscapes, while rectangular fringes are closely related to aquatic environments [11]. In terms of locomotor performance, the function of toe fringes may be related to the type of substrates. For example, there was no significant difference in maximum sprint speed and acceleration on rubber substrates before and after removal of the toe fringes, while locomotor performance on sand substrates decreased significantly (nearly 15%) after removal of toe fringes [12]. These studies suggest that toe fringes are suitable for a test of the relationship between the morphology and environment and trade-offs between sand burial and locomotor performance.

The relationship between sand burial performance and substrate in lizards may be related to substrate properties. As a typical granular substrate, sand is not constant in size, shape, density, coefficient of friction of the particles, and the moisture content between them [13]. This leads to substrate heterogeneity, even if the site of movement only exists on the sand substrate. Although sand has complex substrate properties, some physical properties are not difficult to quantify e.g. median sand grain diameter and angle of stability [14]. The size of the substrate particles provides an indication of the differences between different sand substrates and bulk densities in relation to particle shape [14]. The angle of stability, which is the angle at which the substrate is about to slide as the slope changes [15, 16], reflects not only the stability of the substrate but also the critical state at which the substrate changes from solid to fluid [16].

Substrates influence locomotor performance in different lizard species, but the effects vary considerably in degree, likely reflecting trade-offs in the natural environment as animals switch locomotion on different substrates [17]. The locomotor performance of a generalist lizard is less strongly influenced by substrate type, such as in *Tropidurus torquatus* [18], *Eremias arguta* [19], and *Phrynocephalus helioscopus* [20]. Rhoptropus can maintain its ability to move at high speeds, even in the face of a switch in different terrestrial substrates, consistent with the jack-of-all-trades hypothesis [20, 21]. The relatively wide distribution of these lizard habitats is consistent with the habitat breadth hypothesis [22]. In contrast, some specialist lizards rely on physiological plasticity and morphology to adapt to changing environmental conditions. Specifically, the significant different performance of locomotion on different substrates is consistent with the home field advantage hypothesis [20, 23, 24]. Consideration of the physical properties of substrates facilitate an improved understanding of morphological functions [25].The effect of the properties of the locomotor substrate on their locomotor function is more pronounced among small tetrapods [26–28]. In some cases, animals use the properties of the granular substrate to enhance their locomotor performance. Some lizards use the fluidity of the sand to facilitates sand swimming [7]. Below the surface, instead of using their limbs excessively for propulsion, lizards overcome resistance to movement through body fluctuations [29].

Unlike sand swimming, it has been observed in the field that when sand burial behaviour occurs, the lizard uses rapid limb movement to drive regular fluctuations of the body to bury itself in the sand, with little lateral displacement occurring during the sand burial movement [8]. It is not known whether lizards use sand characteristics to improve their sand burial performance.

*Phrynocephalus mystaceus* is a classical sand-habitat (e.g., semi-fixed dunes) species which widespread in central Asia, including northwest of China [30]. It is the largest and most primitive species of *Phrynocephalus*, which is a genus of toad-headed agama lizards [31]. They have well-developed triangular toe fringes, has evolved rapid sand burial behaviour, and is well suited as a model organism for studying the relationship between morphology, function, and substrates. Although it has been shown that the toe fringes function of *P. mystaceus* was mainly related to sand burial behaviour, the changes in its function under different substrates have not been explored at the substrate level. Therefore, in this study, we performed experimental removals of toe fringes of a sand-dwelling lizard, *P. mystaceus*, to tested the effect of toe fringes on sand burial performance on different sand substrates and to verify the following hypotheses:

1. Assuming that the sand burial performance is related to substrate properties, we predict that whether or not the toe fringes are removed, the angle of stability is the main factor influencing performance. i.e. the sand burial performance is related to the size of the angle of stability.
2. Assuming that toe fringes are a product of adaptation to sand substrate, we predict that sand burial performance on different sand substrates in the uncut toe fringes state is different, which in line with the prediction of the home-field advantage hypothesis, whereas after removal of the toe fringes, performance on different sand substrates is similar, which in line with the prediction of the jack-of-all-trades hypothesis.
3. Assuming that toe fringes function as an adaptation to drifting sand, we predict that compared to individuals with removed toe fringes, individuals with uncut toe fringes would have better sand burial performance on the highly mobile sand.

## Methods

In July 2018, *P. mystaceus* individuals (n = 9) were collected (Animal protocol number: 2017012) by hand from the Tukai Desert (N43° 55′–44° 01′N, 80° 43′–80° 51′E). Individuals were selected that were in good condition and were taken back to the Zoology Laboratory of Xinjiang Agricultural University, where they were housed individually in plastic terraria (30 × 20 × 20 cm, length × width × height). The plastic terraria were covered with 5 cm of fine sand collected from the original *P. mystaceus* habitat, with a 60 W bulb suspended at one end as a heat source for thermo-regulation (from 9 am to 7 pm). *Tenebrio molitor* larvae and water supplemented with calcium and vitamins were provided to ensure that the animals received a full complement of nutrients. Animals were allowed one week after arrival to acclimatize to their new conditions before the experimental trials commenced. All the tests were completed within two weeks.

We measured the snout–vent length (SVL), head length (HL), head width (HW), head depth (HD), mouth breadth (MB), axilla–groin length (AG), abdominal width (AW), tail base width (TBW), forelimb length (FLL), hindlimb length (HLL), tail length (TL) and the lengths of all five digits (I–V) on the ventral side of the right *manus* and *pes* using digital callipers were accurate within 0.1 mm [32, 33], and calculated the toe length ratio of 2D: 4D. The claws were excluded from the digit measurements. The mass was recorded using an electronic balance (NS325-200B, Jiangsu, CHN) to the nearest 0.01 g. To avoid measurement errors, measurements were taken by the same person (the first author) on different individuals.

Toe fringes were quantified and the standardized according to the following characteristic traits: TFN is individuals’ total number of toe fringe in one individual and TFL is individuals’ total max length of each toe fringe (Figure S1a), based on the published measurement procedure and method [3]. This is demonstrated by the area of all toes (TFA) in the uncut state (Figure S1a), the area of all lateral fringes plus the area of all toes in the unilateral cut state, and the area of all toes in the bilateral cut state. The toe fringe area was measured three times. We took pictures of the fringe characteristics with a Canon digital camera and measured and analyzed the data using Image Pro Premier 6.0 software [34].

### Determining the substrate properties

**Fig. 1:**
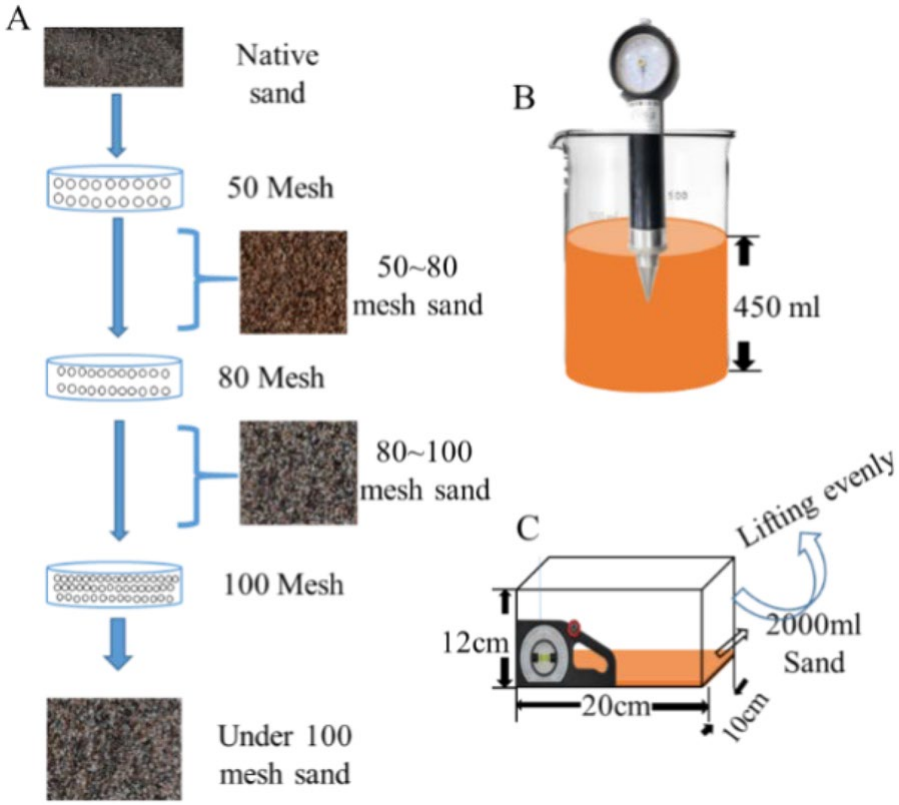
Diagram of substrate property measurements. (**A**: Sifting through sieves for different sand grain sizes including: 0.355–0.200 (50–80 mesh), 0.200–0.150 (80–100 mesh), 0.160–0.196 (native sand) and less than 0.150 (below 100 mesh); **B**: Measurement of sand compactness; **C**: Determination of the sliding stability angle of sand

Although the ecological substrate particle size of the *P. mystaceus* was less studied, we referred to the ecological substrate particle size (0.170–0.375 mm) from *Uma* of the family Iguanidae, which has a series of elongate valvular scales fringing the lateral edges of the toes as well as sand burial behaviour [35]. We passed the native sand substrate through 50, 80 mesh, and 100 mesh sieves and screened four sand substrate types with particle sizes of 0.355–0.200 mm (50–80 mesh), 0.200–0.150 mm (80–100 mesh), 0.160–0.196 mm (native sand), and less than 0.150 mm (below 100 mesh) (Fig. 1a). We measured the density (g/cm^3^) of each substrate by measuring a volume of 100 ml using volumetric bottles, and then weighed the substrate to the nearest 0.1 g using an electronic balance (JM-B, JIMIN, Yutao, Zhejiang, CHN) and divided the mass by the volume. To measure the compactness of the sand substrate, we used a pointer-type soil hardness meter (TYD-1, Zhejiang, CHN), which was inserted vertically into a beaker with a capacity of 500 ml to measure the firmness of the different sand substrates (450 ml; kg/cm^2^; Fig. 1b). To measure the angle of stability, we placed 2,000 ml of substrate in a 20 × 10 × 12 cm (length × width × height) ) plastic container (Fig. 1c), fixed the slope meter (Wenzhou, Shanghai, CHN) to the plastic container, and kept it at the same level. A section was lifted at a constant speed; Until the sand surface creates a slide. The angle at which this occurs is the angle of stability [14]. The substrate density, compactness, and angle of stability were measured for four replicates, namely native sand, 50–80 mesh, 80–100 mesh, and below 100 mesh, and this was repeated ten times for each sand substrate [14].

### Determination of sand burial performance

The sand burial performance was measured in a tank (35 × 20 × 20 cm, length × width × height), and the bottom was covered with 10 cm of sand (native sand, 50–80 mesh, 80–100 mesh, below 100 mesh). We used a brush to stimulate the tail, which caused the lizards to dive into the sand. The entire process was recorded with a digital camera (Canon EOS 7d, FPS:50), and sand burial behaviour was analysed through video playback. Complete sand burial behaviour was also divided into three repetitions, namely with uncut fringe, unilateral cut, and bilateral cut (The same individuals repeated three times, N = 9, Figure S1b). To avoid the effect of physical trauma caused by the removal the fringe on the sand burial performance of the lizard, we paid close attention to the physical condition of the lizard when removing the fringes and care was taken during fringe removal.

The sand burial performance of *P. mystaceus* was categorized into the sand burial ability score and the sand burial time score based on the duration of the sand burrowing process and the degree of sand burial. The comprehensive score was the sum of the two score types, The burial time and burial ability were scored independently and the comprehensive score was the sum of the two scores. The criteria for rating these two scores are listed in Table 1. to avoid perceived errors, two scores were recorded by the same person watching the sand burial video based on the criteria in Table 1.

**Table 1:** Sand burial ability score and sand burial time score rating table

Scoring Standards	5	4	3	2	1	0
Sand burial ability score	Fully buried	Tail not buried	Head not buried	Most of the body not buried	With sand-burial tendency, not buried in the sand	No sand- burial tendency
Sand burial time score (s)	0–0.5	0.5–1.0	1 –1.5	1.5 –2.0	> 2.0	No sand- burial tendency

The following sand burial indicators were recorded: NHS (number of hind-limb swings) was calculated by Eq. 1, including THS (time of hind-limb swings) was calculated by Eq. 2 and FHS (frequency of hind-limb swings) was calculated by Eq. 3 [3].

1 $${\text{NHS}} = {\text{TNHS}}/{\text{BAS}}$$

2 $${\text{THS}} = {\text{TBT}}/{\text{BAS}}$$

3 $${\text{FHS}} = {\text{NHS}}/{\text{THS}}$$

where TNHS: individuals’ total number of hind limb swings during sand burial; BAS: the score of sand burial ability; TBT: individuals’ total sand burial time.

### Statistical analyses

Kolmogorov–Smirnov tests were used to detect normality. We log-transformed the variables to minimize heterogeneity where necessary [36]. We used a linear mixed-effects model to test the effects of different substrates and different toe fringes status on the sand burial performance of *P. mystaceus*, as well as the interaction between the two factors, and the results showed that the interaction was significant, and therefore the two factors were further analyzed separately (Table S2). We performed a repeated measures ANOVA to examine the differences in sand burial performance of the different states on the four sand substrate types using paired-sample t-tests (multiple comparisons). We corrected for multiple comparisons using the Benjamini–Hochberg method [37]. We tested whether substrates of different particle sizes differed in density, compactness, and angle of stability using ANOVA [14]. We also examined the significance of the difference in the slope of sand burial performance in different states (uncut, bilateral-cut) on different substrate properties (stability angle), with the stability angle as a covariate.

Multiple regression is a suitable test for estimating the effects of morphological characteristics on the biological functions of sand burial. However, SVL, FLL, HLL, TL, mass, and other morphological indicators tended to display high levels of multicollinearity, which can invalidate multiple regression analyses. Therefore, the Farrar–Glauber test [38] was used to assess whether extensive multicollinearity existed among the traits. Significant multicollinearity occurred for all nine log-transformed traits (Farrar chi-square = 1.059^e+18^ > 1,000). Compared with other analysis methods including ridge regression, principal component regression, and least squares, LASSO regression has the smallest prediction error and plays a good role in the high-dimensional multicollinearity problem [39]. Therefore, to avoid the effect of multicollinearity between morphologies on sand burial performance, we used the LASSO regression to test the relationship between morphology and function [39, 40]. We used all the morphological and substrate traits as independent variables and the comprehensive score as the dependent variable. We censored the indicators related to sand burial performance using LASSO regression (R, glment). Path analysis allows the simultaneous influences of several variables on the dependent variable to be examined. Examining the simultaneous effects of several variables on the dependent variable provides a clearer picture of the relationship between variables [41]. Therefore, we conducted a path analysis implemented in Amos (v.24.0) to assess the morphological characteristics selected during sand burial to explore their internal relationships [3]. All the rest analyses were conducted using R v. 4.1.1. [42].

## Results

### Sand burial performance

#### Sand burial performance of *P. mystaceus* in different states

**Fig. 2:**
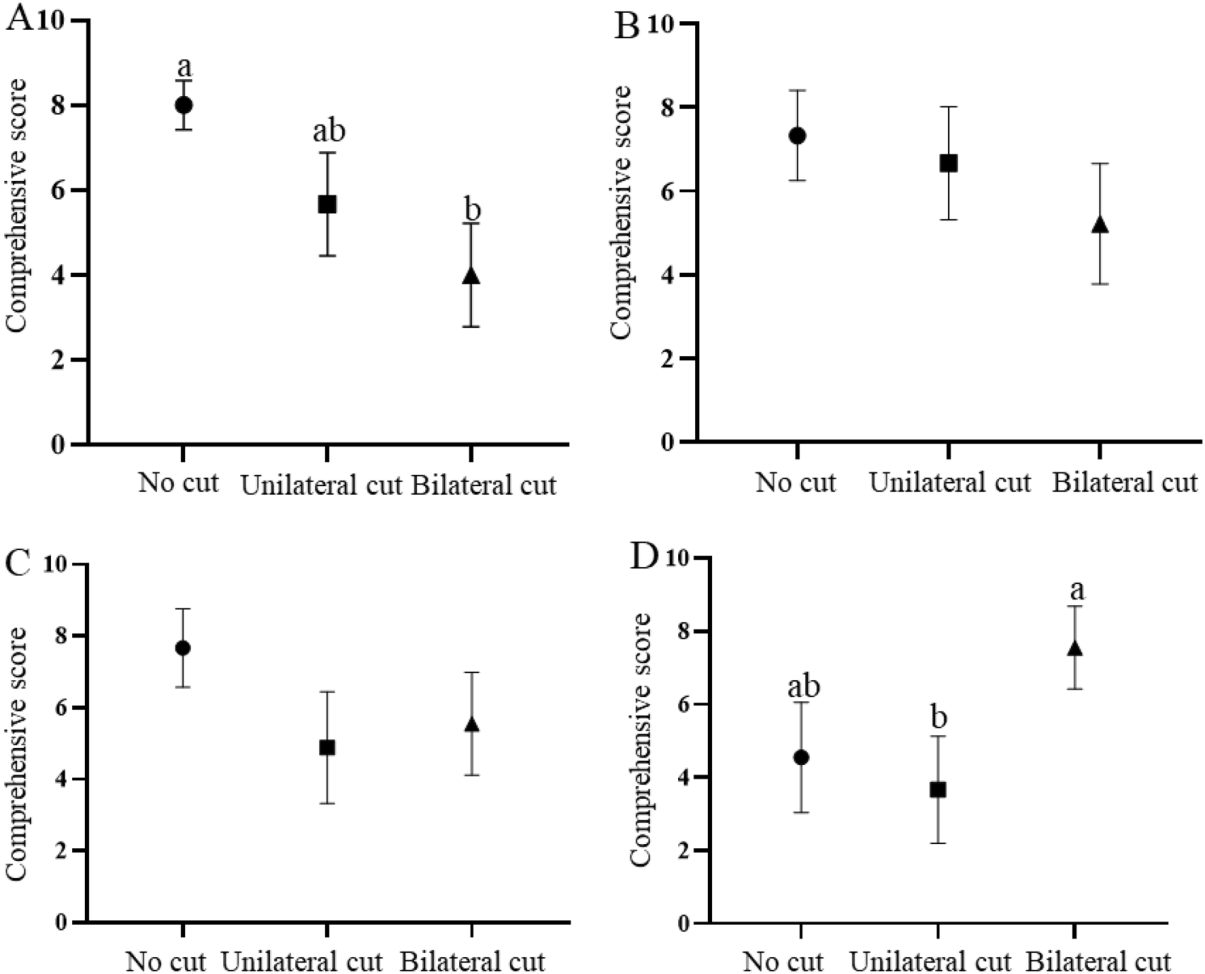
Scores of sand burial in different states for *Phrynocephalus mystaceus.* **A**: native substrate; **B**: 50 mesh to 80 mesh substrates; **C**: 80 to 100 mesh substrates; **D**: below 100 mesh substrate. Notes: Different letters indicate significant differences at the *P* < 0.05 level

**Fig. 3:**
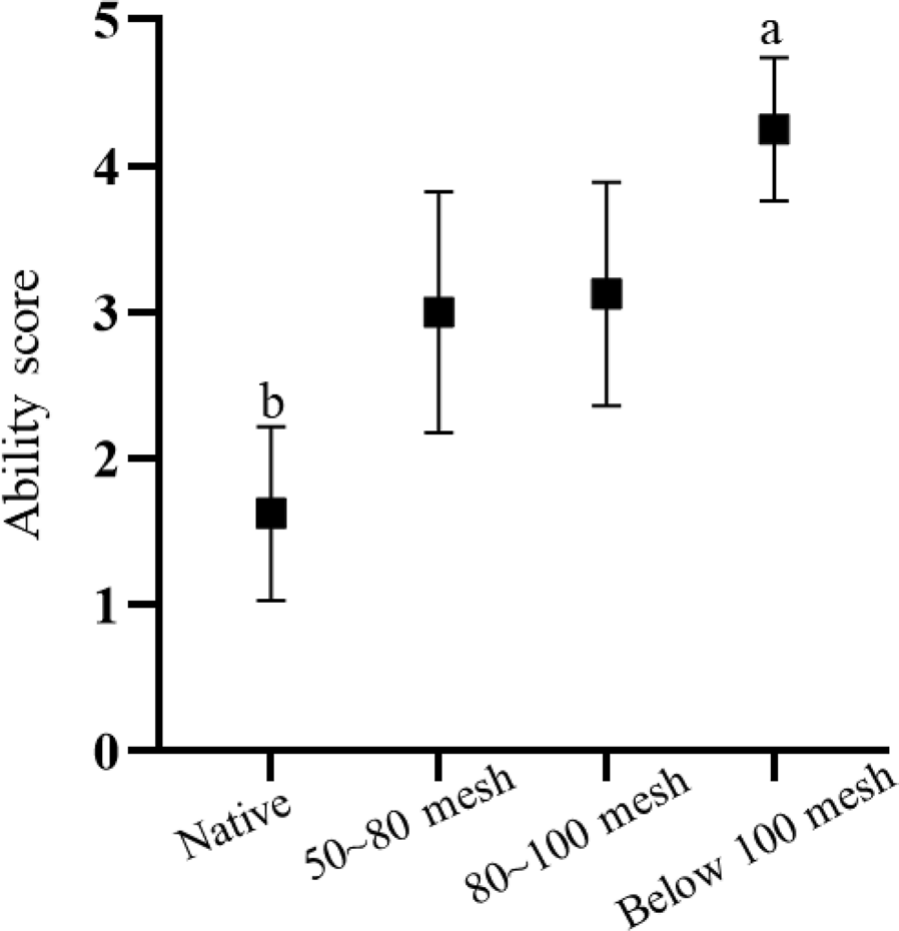
Sand burial performance of *Phrynocephalus mystaceus* on different substrates after removal of bilateral toe fringes. Note: Different letters indicate significant differences at the *P* < 0.05 level

We found significant differences in the comprehensive scores of *P. mystaceus* on the native substrate in different toe fringe states (F_2,24_ = 3.893, *P* = 0.042) (Fig. 2a). The sand burial performance of *P. mystaceus* was significantly higher in the uncut toe fringe state than in the bilateral toe fringe removal state (t = 3.179, *P* = 0.039). However, there was no significant difference in the other states, namely uncut vs. unilateral cut (t = 2.111, *P* = 0.102) or unilateral cut vs bilateral cut (t = 0.902, *P* = 0.386) (Fig. 2a). In terms of 50 mesh to 80 mesh and 80 to 100 mesh sand substrates, there were no significant differences in the performance of sand burial in different toe fringe states (F_2, 24_ = 3.669, *P* = 0.062, Fig. 3b; F_2, 24_ = 1.830, *P* = 0.209, Fig. 2b and c). For the sand substrate below 100 mesh, the comprehensive scores for the unilateral cut state were significantly lower than those for bilateral toe fringe removal for *P. mystaceus* (t =  − 3.026, *P* = 0.048, Fig. 2d). The rest of the substrates did not significantly differ (no cut vs. bilateral cut, t =  − 1.701, *P* = 0.190; no cut vs. unilateral cut, t = 0.574, *P* = 0.582).

#### Sand burial performance of *P. mystaceus* in different substrates

Under the uncut state and unilateral cut state, there were no significant differences in the performance of sand burial on different substrates (*P* > 0.05 in both cases, Figure S2).

Under the bilateral cut state, there were no significant differences in the comprehensive scores and time scores of sand burial on different substrates (comprehensive scores: F_3,28_ = 5.679, adjusted *P* = 0.054, time scores: F_3,28_ = 3.714, *P* = 0.051, Figure S2). In terms of ability score, we found significant differences in the performance of sand burial substrates (F_3,28_ = 6.876, *P* = 0.002), and multiple comparisons revealed that the ability score of *P. mystaceus* on native substrates was significantly smaller than that on 100 mesh substrates (t =  − 4.020, *P* = 0.030; Fig. 3).

**Fig. 4:**
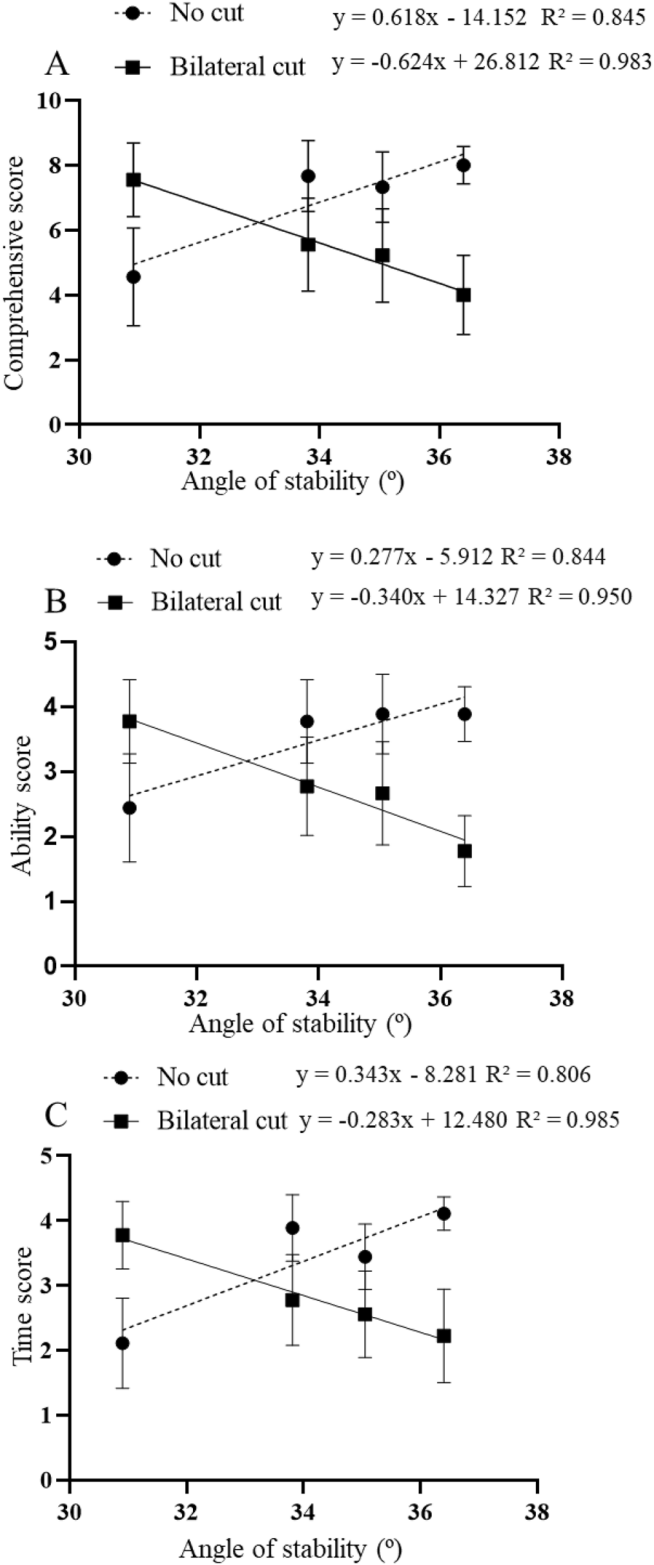
Regression of sand burial performance and stability angle in the uncut and bilateral cut states of *Phrynocephalus mystaceus* (**A** Comprehensive scores; **B** Ability score; **C**: Time scores)

With an increase in the stability angle, there was a significant difference in the slope of sand burial performance for the uncut and bilateral cut states (comprehensive scores: F_1, 70_ = 8.987, *P* = 0.004, Fig. 4a; ability score: F_1, 70_ = 7.347, *P* = 0.008, Fig. 4b; time score: F_1, 70_ = 9.603, *P* = 0.003, Fig. 4c).

### Substrate properties

**Fig. 5:**
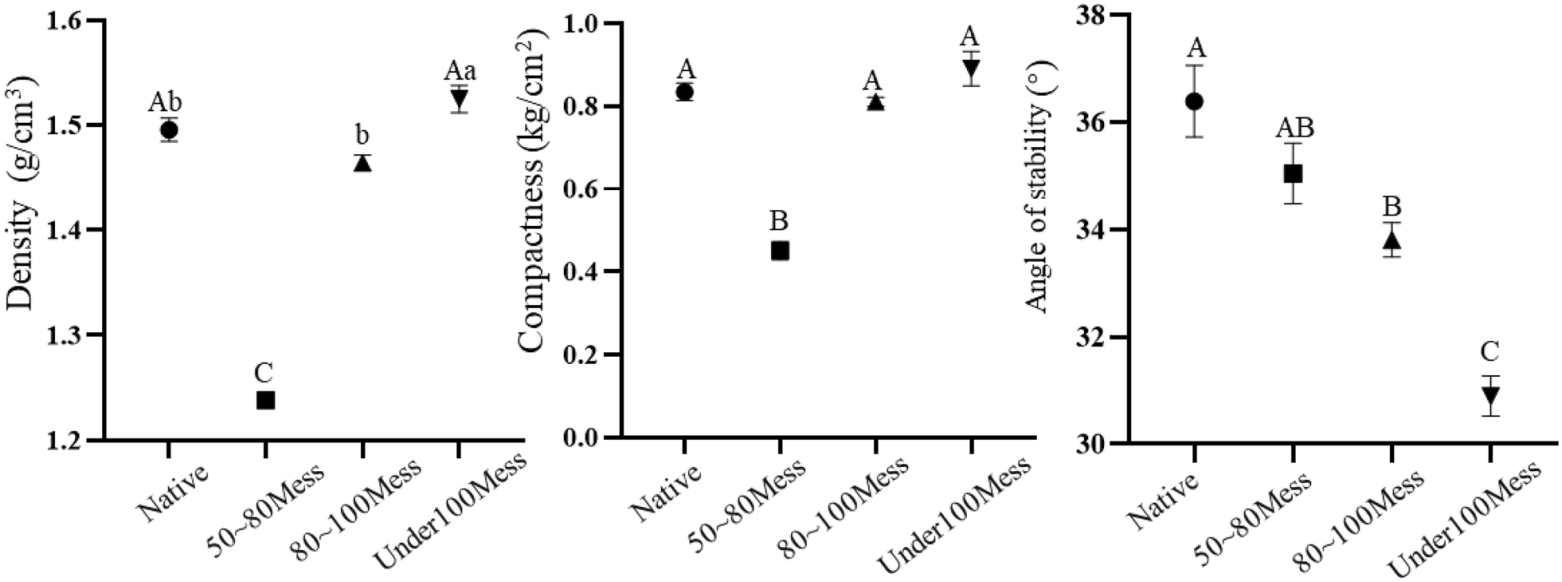
Properties of different substrates. Note: Different uppercase letters indicate extremely significant differences at *P* < 0.01 level; different letters indicate significant differences at *P* < 0.05.

There were significant differences in density among the four substrates (F_3, 36_ = 171.275, *P* = 0.000). Further analysis showed that the 50 mesh to 80 mesh sand substrates were significantly smaller than the remaining three substrates (*P* = 0.000, Fig. 5a; Tables S3 and S4). The density of sand substrates below 100 mesh was significantly higher than the density of sand between 80 and 100 mesh (*P* = 0.020, Fig. 5a; Tables S3 and S4).

There were highly significant differences in compactness among the four substrates (F_3, 36_ = 57.849, *P* = 0.000). This was demonstrated by the 50 mesh to 80 mesh sand substrates being significantly smaller than the remaining three substrates (*P* = 0.000, Fig. 5b; Tables S3 and S4).

The four substrates were significantly different in terms of angle of stability (F_3, 36_ = 21.696, *P* = 0.000), as shown by the angle of stability of the sand substrate below 100 mesh being significantly smaller than that of the remaining three substrates (native: *P* = 0.000; 50–80 mesh, *P* = 0.000; 80–100 mesh, *P* = 0.002, Fig. 5c; Tables S3 and S4). The stability angle of the native sand substrate was significantly larger than that of the 80 mesh to 100 mesh (*P* = 0.007; Tables S3 and S4).

### Effect of morphology and substrate characteristics on the function of sand burial

**Fig. 6:**
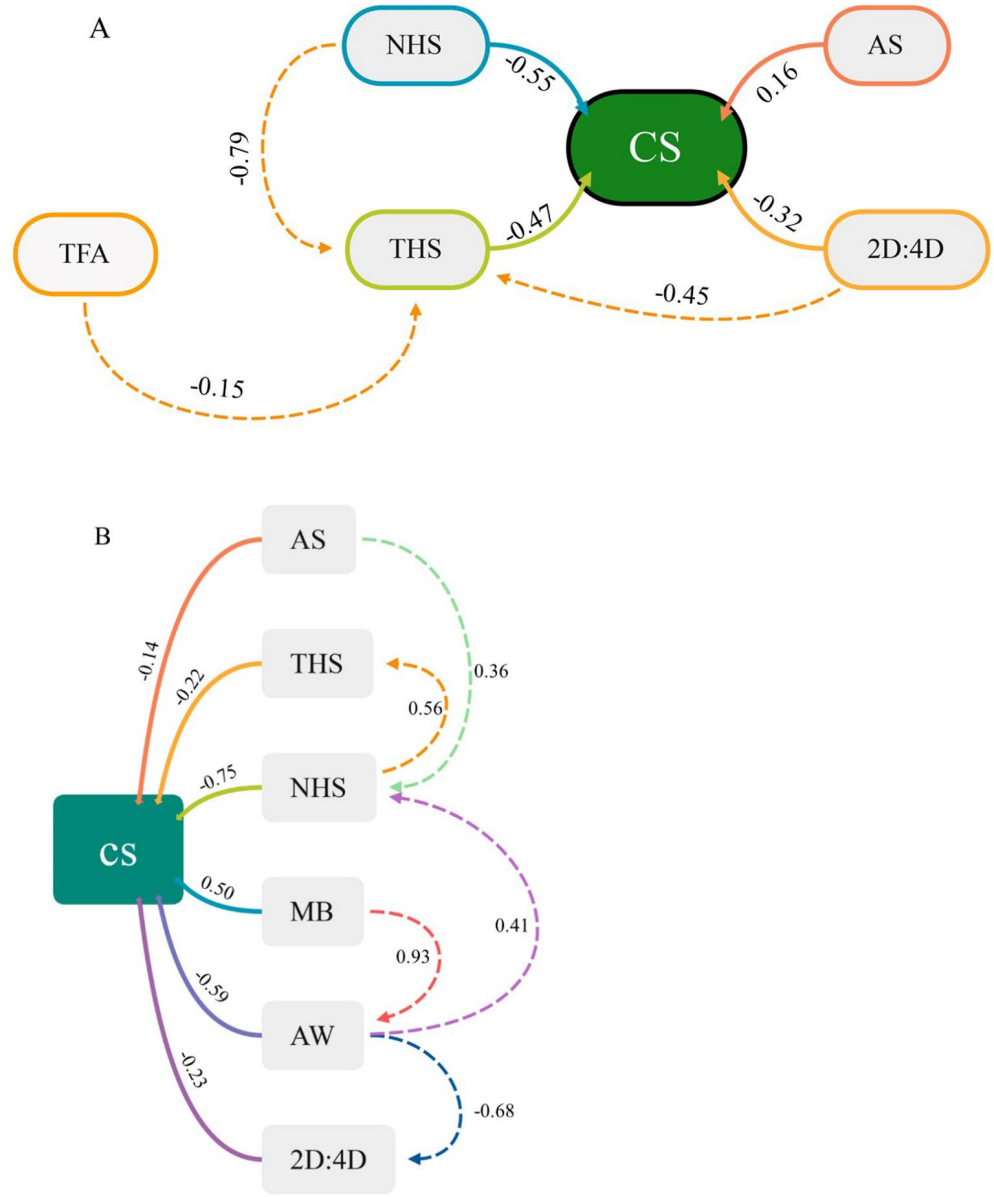
Path analysis diagram of sand burial of *Phrynocephalus mystaceus*. Note: This path model examines how sand burial is affected by the morphology and different substrates (CS: comprehensive score; NHS: number of hind-limb swings; THS: time of hind-limb swings; TFA: the total area of all toes; AS: angle of stability; 2D:4D: toe length ratio of 2D: 4D; MB: mouth breadth; AW: abdominal width). **A**: no cut; **B**: bilateral cut. The solid lines represent the relationship between the different indicators and the comprehensive score, the dashed lines represent the relationship between the individual indicators. Numbers next to the path represent the relative contributions of different substrates during sand burial.

Under the uncut toe fringe state, the LASSO regression results showed that AS, 2D: 4D, TFL, NHS, and THS were the main factors affecting the comprehensive sand burial score (Table S5). The results of the path analysis showed that NHS, AS, THS, and 2D: 4D could directly affect the sand burial performance of *P. mystaceus* (Fig. 6a) and were significantly correlated (Table 2). Only AS was positively affected. TFA can also indirectly affect the sand burial performance of *P. mystaceus* through THS. Similarly, NHS and 2D: 4D can also indirectly affect the sand burial performance of *P. mystaceus* through THS (Fig. 6a) and cause significant effects (Table 2).

**Table 2:** Regression weights of the path analysis result about sand burial performance of *Phrynocephalus mystaceus*

States	Path label			Estimate	S.E	C.R	*P*
Uncut	THS	←	TFA	− 0.178	0.089	− 1.989	0.047*
	THS	**←**	NHS	0.607	0.057	10.703	0.000***
	THS	**←**	2:4D	− 1.513	0.244	− 6.189	0.000***
	CS	**←**	AS	12.012	3.933	3.055	0.002**
	CS	**←**	NHS	− 4.486	0.939	− 4.778	0.000***
	CS	**←**	24D	− 11.329	2.812	− 4.029	0.000***
	CS	**←**	THS	− 4.996	1.363	− 3.666	0.000***
Bilateral cut	AW	**←**	MB	1.751	0.145	12.071	0.000***
	NHS	**←**	AS	3.66	1.76	2.079	0.038*
	NHS	**←**	AW	0.993	0.417	2.381	0.017*
	THS	**←**	NHS	0.569	0.174	3.281	0.001**
	2D:4D	**←**	AW	− 0.262	0.058	− 4.503	0.000***
	CS	**←**	AS	− 12.053	4.3	− 2.803	0.005**
	CS	**←**	MB	18.936	4.716	4.015	0.000***
	CS	**←**	AW	− 11.85	2.68	− 4.422	0.000***
	CS	**←**	NHS	− 6.116	0.527	− 11.608	0.000***
	CS	**←**	THS	− 1.781	0.454	− 3.926	0.000***
	CS	**←**	2D:4D	− 11.633	3.294	− 3.531	0.000***

Estimate represents the regression coefficient for each path (CS: comprehensive score; NHS: number of hind-limb swings; THS: time of hind-limb swings; TFA: the area of all toes; AS: angle of stability; 2D:4D: toe length ratio of 2D: 4D; MB: mouth breadth; AW: abdominal width). The standard errors of the estimates (Std. Error), t-value, and probability (P) were estimated using path analysis in Amos (v.24.0). Significance levels: *P < 0.05, **P < 0.01, ***P < 0.001.

In the unilateral cut toe fringe state, FLL and TFA were positive factors affecting the comprehensive sand burial score. In comparison, NHS was a negative factor (Table S5).

The MB was the only positive impact indicator in the bilateral cut toe fringe state. In contrast, AS, AW, 2D: 4D, NHS, and THS were the main negative factors (Table S5). The path analysis showed that AS, THS, NHS, MB, AW, and 2D: 4D could directly affect the sand burial performance of *P. mystaceus* (Fig. 6b) and were significantly correlated (Table 2). In contrast with the uncut state, the role of AS changed from positive to negative after the removal of the bilateral toe fringes.

## Discussion

The physical properties of granular substrates can be used by animals to enhance their locomotor functions. Sandfish (*Scincus scincus*) can swim in sand without using their limbs [7]. Sand dunes are well-aerated and provide sufficient oxygen, and the slippery surface is looser for lizards to bury themselves in the sand [5]. Different substrate properties affect the conditions under which these behaviours occur. Our results show that the angle of stability of the native sand substrate with non-uniform particle size under natural conditions is larger than that of the three remaining uniform substrates. It is significantly larger than that of sand substrates with 80–100 mesh and less than 100 mesh. In the case of homogeneous substrates, the smaller the particles, the smaller is the angle of stability. Given that there are gaps in the granular substrate and in the inhomogeneous substrate, the smaller particles can fill these gaps, making the substrate more stable [43]. The relationship between the morphology, substrate characteristics, and sand burial function varies with the state of the toe fringes. The results of the LASSO regression and the path analysis showed that the sand burial performance of *P. mystaceus* was influenced by the angle of stability when the toe fringes were not removed (Table S5) and showed a significant positive correlation (Fig. 6a and Table 2). After cutting the bilateral toe fringes, we found that the effect of the angle of stability during sand burial was the opposite of that when the toe fringes were not cut (Table S5 and Fig. 6b). These results are in line with the prediction of our hypothesis 1 that the angle of stability is the main factor influencing sand burial performance.

We found that the sand burial performance of *P. mystaceus* was not affected by the substrate in the uncut and unilateral cut toe fringe states (Figure S2a and d). Interestingly, when the bilateral toe fringes were removed, the sand burial ability scores of the lizard were significantly higher on sand substrates with less than 100 mesh than on native sand substrates (Fig. 3). These results are contrary to our hypothesis 2. Sand burial is a specialized anti-predation strategy because it can only occur on sand substrates [5]. If the sand substrate is refined into multiple particle size substrate types, sand burial performance of removal bilateral toe fringes is consistent with the home field advantage hypothesis (best performance on the most familiar substrate) [20]. These results indicate that the sand burial performance was influenced by substrate characteristics after the removal of the bilateral toe fringes and that it performed better on substrates with smaller particles. This may be related to the mobility of the different sands, and substrates with medium particles are more conducive to lizard movement [14]. Due to climate changes during the Cenozoic, including the ongoing aridification of central Eurasia [44–46], the common ancestor of *Phrynocephalus* probably preferred sandy substrates with the inclusion of clay or gravel [47]. The origin of toe fringes and sand-burial behaviour may have been an adaptation to coarser gravels, and later, due to habitat changes, the sand-burial function of toe fringes was weakened on fine sand substrates (Fig. 2D and 3). On the other hand, developed toe fringes facilitate the movement of lizard on highly mobile fine sand [12].

The relationship between toe fringe function as an adaptation and substrate may not be one-to-one. On the native sand substrate, the sand burial performance with bilateral toe fringes was significantly higher than in the removed bilateral state (Fig. 2a). However, this relationship was reversed with substrate change, and the sand burial performance improved in the sub-100 mesh sand substrate (Fig. 2d). TFL can positively influences sand burial performance (Table S5) and TFA can indirectly influence sand burial through THS (Fig. 6a and Table 2), suggesting that toe fringes can be applied to less mobile substrates. We found a significant negative correlation between THS and TFA, suggesting that when toe fringes are involved, they can reduce hind limb oscillation and save energy, which is similar to the results of related studies [3]. It has been shown that the locomotor performance of lizards was significantly negatively correlated with sand burial after the loss of toe fringes [3], so we hypothesis that on coarse sand, toe fringes can help lizards to bury sand and conserve energy, while on fine sand, the function of toe fringes may be correlated with the locomotor, allowing it to perform similarly well regardless of the sand substrate, thereby improving species fitness.

The smaller the stability angle, the more mobile the sand substrate, the easier it is for the limbs to enter the sand, and the less the hind limbs swing. This also caused a significant difference in the sand burial performance between the different toe fringe states as the stability angle increased (Fig. 4). In particular, the ability to bury sand after removal of the bilateral toe fringes scored better on substrates with high mobility (below 100 mesh) (Fig. 3), contrary to our hypothesis 3, where the role of toe fringes function may not be apparent on substrates with high mobility. Sand burial and running on sand are two completely different types of anti-predatory behaviour. In the case of running, the primary role of the substrate is to provide support and a stable, flat surface. Therefore, the stabe friction of the substrate is likely the main influencing factor [14]. The roughness and texture of the substrate surface affect the ability to climb and run [28, 48–50], and a grippy substrate increases the average maximum sprint speed of the lizard [51]. Although sand burial behaviour is similar to that of digging [3], the mobility of the substrate is particularly important. We found that the smaller the substrate particles, especially sand substrates below 100 mesh, the smaller the angle of stability. This corresponds to greater mobility, and a more mobile substrate means that it is easier to bury itself in sand. This is why sand burial performance is significantly better on substrates below 100 mesh than on native sand substrates. On the other hand, locomotion on highly mobile substrates requires a larger contact area for balance and friction, and the TFA increases the contact area of the lizard's foot with the substrate, suggesting that the role of the toe fringes may be relevant to locomotion on highly mobile substrates.

In addition to toe fringes, the digit ratio has also been shown to correlate with anti-predatory behaviour [52]. It has been shown in studies of human locomotor performance that individuals with low digit ratios tend to excel in physical performance, especially in endurance-related sports [53]. Our results have shown that 2D: 4D was significantly and negatively correlated with sand burial performance in *P. mystaceus* (Fig. 6a and b and Tables 2 and S4), regardless of whether the toe fringes were removed. Individuals with lower digit ratios performed better in sand burial. The fact that 2D: 4D is often thought to be associated with androgens [52–56] also suggests that the sand burial behaviour of *P. mystaceus* may also be sex-linked and needs to be further explored.

Overall, the results of toe fringe morphology and function emphasize the importance of habitat use for sand living lizards. On natural substrates, the performance of lizards can predict habitat use [57]. Future research should focus on the function of toe fringe across species and reveal the mechanisms of different resource use in combination with ecological substrates.

## Conclusions

The function of the toe fringes of *P. mystaceus* varies according to substrate properties. The effect of toe fringes on the sand burial performance gradually decreased with a decrease in the stability angle of the substrate. This also indicates that the sand burial function of toe fringes may not be suitable for highly mobile sand substrates. In this case, it remains to be further tested whether the function of toe fringes is more important for running on sand.

## Supplementary information

Supplementary material 1

## Data Availability

The authors declare that all data supporting the findings of this study are available within the article [and its supplementary information files].

## Abbreviations

SVL: Snout–vent length, from snout to vent
HL: Head length, from rostrum to occipital sinus
HW: Head width, measuring widest part of the head
HD: Head depth, measuring highest part of the head
MB: Mouth breadth, from left mouth point to right mouth point
AG: Axilla–groin length, from edge of the forelimb to edge of hindlimb
AW: Abdominal width, straight-line distance from the widest part of the abdomen
TBW: Tail base width, maximum width at base of tail
FLL: Forelimb length, from forelimb insertion to the tip of the longest finger
HLL: Hindlimb length, from hindlimb insertion to the tip of the longest toe
TL: Tail length, from vent to the tip of the tail
2D: 4D: calculated the toe length ratio of 2D: 4D
TFA: The total area of all toes
TFL: Individuals’ total max length of each toe fringe
TFN: Individuals’ total number of toe fringe
TNHS: Individuals’ total number of hind limb swings during sand burial
BAS: The score of sand burial ability
TBT: Individuals’ total sand burial time
NHS: Individuals’ total number of hind limb swings during sand burial divided by the score of sand burial ability
THS: Individuals’ total sand burial time divided by the sand burial ability score
FHS: Individuals’ NHS divided by THS
AS: Angle of stability
