# Supplementary material for: Are toe fringes important for lizard burying in highly mobile sand?
Source: Front Zool. 2024 Sep 30;21:25. doi: 10.1186/s12983-024-00546-y (PMC11440683; doi:10.1186/s12983-024-00546-y)
Supplement: Supplementary file 1 — Supplementary material 1 [file 12983_2024_546_MOESM1_ESM.docx]

**Supplementary Figures and Tables**


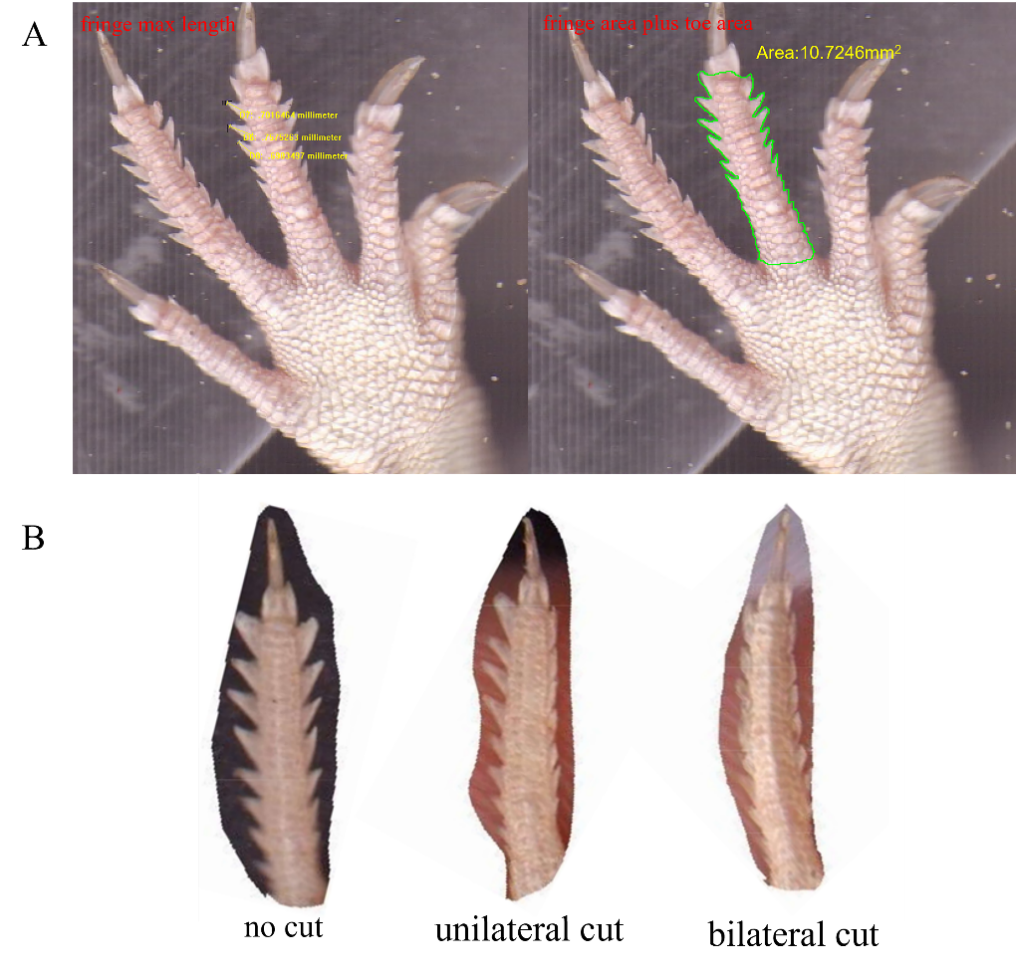


**Figure S1** Diagram of the measurement and removal of toe fringes of the *P. mystaceus.* A: toe fringes max length and toe fringes area plus toe area measurement of the third finger; B: Pictures of fringe under three states.


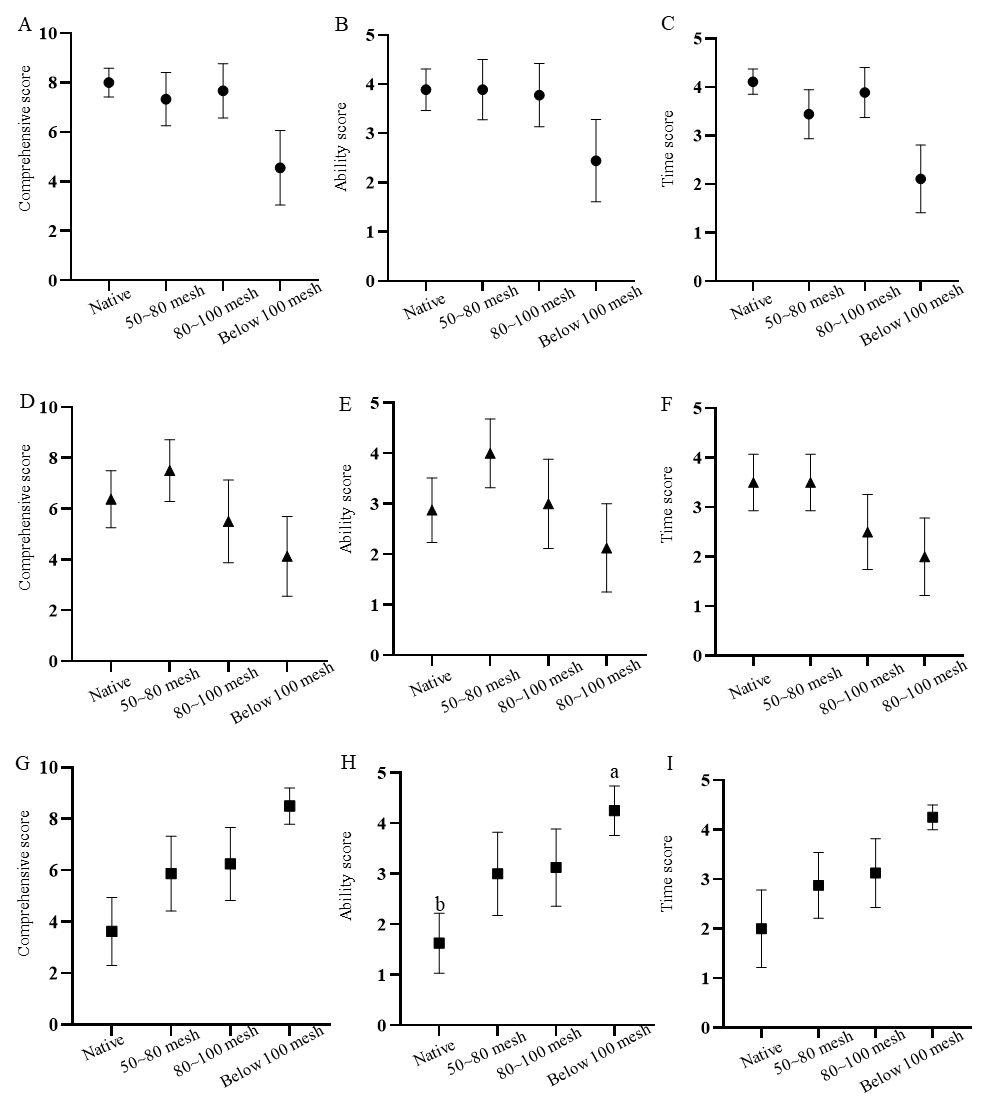


**Figure S2** Sand-burying performance of *P. mystaceus* on different substrates. (●：no cut; ▲:unilateral cut; ■:bilateral cut) Note: Different letters indicate significant differences at the *P* ＜0.05 level

**Table S1** Burying styles in lizard families (from Arnold [8] and Halloy et al. [18])

| Family | Burying mode |  |
| --- | --- | --- |
| Agamidae | Submerge into sand vertically, with an oscillating lateral movement of limbs and later of head and tail (e.g., *Phrynocephalus,* Central and Southwest Asia). |  |
|  |  |  |
| Cordylidae | Swim into sand, limbs folded along body, moving with rapid sinusoidal-like waves |  |
|  |  |  |
| Scincidae | Swim into sand, same description as above (e.g., *Scincus*, North Africa). |  |
| Lacertidae | Dive head first, use all their limbs, flexing head and body laterally, tail lashed, back steps (e.g., *Meroles*, Southwest Africa) |  |
|  |  |  |
| Phrynosomatidae | Dive head first, head twisted about sagittal axis, use mostly hind limbs, forelimbs, placed along body, tail "shimmied", back steps (e.g., *Uma*, North America). |  |
|  |  |  |
|  | Combination of head first diving and vertical sinking with head swung from side to side, followed by hind limbs oscillated from side to side (e.g., *Phrynosoma*, North) America |  |
|  |  |  |

**Table S2** The linear mixed-effects model of Toe fringes and substrates

| Score type | item | *if* | F | *P* |
| --- | --- | --- | --- | --- |
| Comprehensive Score | slope | 1 | 10.439 | 0.001 |
|  | Toe fringes states | 2 | 0.764 | 0.469 |
|  | substrates | 3 | 0.018 | 0.997 |
|  | Toe fringes states*substrates | 6 | 2.413 | 0.033 |
| Ability score | slope | 1 | 244.671 | 0 |
|  | Toe fringes states | 2 | 0.705 | 0.497 |
|  | substrates | 3 | 0.856 | 0.467 |
|  | Toe fringes states*substrates | 6 | 1.839 | 0.101 |
| Time score | slope | 1 | 59.864 | 0 |
|  | Toe fringes states | 2 | 0.76 | 0.471 |
|  | substrates | 3 | 0.074 | 0.974 |
|  | Toe fringes states*substrates | 6 | 2.811 | 0.015 |

**Table S3** Multiple comparisons of substrate properties between different substrates

| Substrate properties | Substrate type | *df* | t | *P* |
| --- | --- | --- | --- | --- |
| Density | native vs 50~80 mesh | 36 | 18.215 | 0.000 |
|  | native vs 80~100 mesh | 36 | 2.217 | 0.166 |
|  | native vs below 100 mesh | 36 | -2.082 | 0.178 |
|  | 50~80 mesh vs 80~100 mesh | 36 | -15.999 | 0.000 |
|  | 50~80 mesh vs below 100 mesh | 36 | -20.297 | 0.000 |
|  | 80-100 mesh vs below 100 mesh | 36 | -4.299 | 0.002 |
| Compactness | native vs 50~80 mesh | 36 | 10.314 | 0.000 |
|  | native vs 80~100 mesh | 36 | 0.645 | 0.530 |
|  | native vs below 100 mesh | 36 | -1.510 | 0.917 |
|  | 50~80 mesh vs 80~100 mesh | 36 | -9.670 | 0.000 |
|  | 50~80 mesh vs below 100 mesh | 36 | -11.824 | 0.000 |
|  | 80-100 mesh vs below 100 mesh | 36 | -2.154 | 0.234 |
| Angle of stability | native vs 50~80 mesh | 36 | 1.896 | 0.298 |
|  | native vs 80~100 mesh | 36 | 3.637 | 0.007 |
|  | native vs below 100 mesh | 36 | 7.724 | 0.000 |
|  | 50~80 mesh vs 80~100 mesh | 36 | 1.741 | 0.318 |
|  | 50~80 mesh vs below 100 mesh | 36 | 5.828 | 0.000 |
|  | 80-100 mesh vs below 100 mesh | 36 | 4.087 | 0.002 |

**Table S4** Descriptive statistics of substrate properties between different substrates

| Substrate properties | Substrate type | Mean | SE |
| --- | --- | --- | --- |
| Density | native | 1.496 | 0.011 |
|  | 50~80 mesh | 1.238 | 0.007 |
|  | 80~100 mesh | 1.464 | 0.007 |
|  | below 100 mesh | 1.525 | 0.013 |
| Compactness | native | 0.835 | 0.068 |
|  | 50~80 mesh | 0.451 | 0.070 |
|  | 80~100 mesh | 0.811 | 0.033 |
|  | below 100 mesh | 0.891 | 0.131 |
| Angle of stability | native | 36.400 | 2.119 |
|  | 50~80 mesh | 35.050 | 1.787 |
|  | 80~100 mesh | 33.810 | 1.014 |
|  | below 100 mesh | 30.900 | 1.197 |

**Table S5** Effects of morphological characteristics on sand burying score of *Phrynocephalus mystaceus*

| State | Score Type | Traits | Coef |
| --- | --- | --- | --- |
| No cut | Comprehensive Score | AS | 4.510 |
|  |  | 2D:4D | -4.090 |
|  |  | TFL | 1.367 |
|  |  | NHS | -4.974 |
|  |  | THS | -2.592 |
|  | Time Score | MB | -0.239 |
|  |  | THS | -2.048 |
|  | Ability Score | AS | 3.826 |
|  |  | HD | 5.194 |
|  |  | 2D:4D | -3.744 |
|  |  | NHS | -4.622 |
| unilateral cut | Comprehensive Score | FLL | 0.472 |
|  |  | TFA | 2.607 |
|  |  | NHS | -4.238 |
|  | Time Score | THS | -2.980 |
|  | Ability Score | HLL | 4.014 |
|  |  | TFA | 1.839 |
|  |  | NHS | -1.709 |
|  |  | FHS | -3.169 |
| bilateral cut | Comprehensive Score | AS | -15.073 |
|  |  | MB | 6.383 |
|  |  | AW | -2.967 |
|  |  | 2D:4D | -5.147 |
|  |  | NHS | -6.076 |
|  |  | THS | -2.199 |
|  | Time Score | AS | -2.002 |
|  |  | AW | -0.725 |
|  |  | NHS | -1.471 |
|  |  | THS | -0.822 |
|  | Ability Score | THS | -3.459 |
